# Comparative analysis of primer sets for the assessment of clonality in feline lymphomas

**DOI:** 10.3389/fvets.2024.1356330

**Published:** 2024-05-07

**Authors:** Angelika Weyrich, Werner Hecht, Kernt Köhler, Christiane Herden, Manfred Henrich

**Affiliations:** Institut für Veterinär-Pathologie, Justus-Liebig-Universität Giessen, Giessen, Germany

**Keywords:** PCR, antigen receptor rearrangements, clonality, *Felis catus*, lymphoma, cat, PARR

## Abstract

**Introduction:**

Lymphomas are among the most important and common malignant tumors in cats. Differentiating lymphomas from reactive lymphoid proliferations can be challenging, so additional tools such as clonality assessment by PCR are important in diagnosis finding. Several PCR assays have been developed to assess clonality in feline lymphomas. For T-cell lymphomas TRG (T-cell receptor gamma) genes are the preferred target whereas for B-cell lymphomas most primer sets target immunoglobulin heavy chain (IGH) genes. Here we compare commonly used diagnostic primer sets for the assessment of clonality in feline lymphomas under controlled conditions (i.e., identical sample set, PCR setup, amplicon detection system).

**Methods:**

Formalin-fixed and paraffin-embedded samples from 31 feline T-cell lymphomas, 29 B-cell lymphomas, and 11 non-neoplastic controls were analyzed by PCR combined with capillary electrophoresis.

**Results and discussion:**

We show that the combination of the primer sets published by Weiss et al. and Mochizuki et al. provided the best results for T-cell clonality, i.e., correctly assigns most populations as clonal or polyclonal. For B-cell clonality, the combination of the primer sets by Mochizuki et al. and Rout et al. gave the best results when omitting the Kde gene rearrangement due to its low specificity. This study rigorously evaluated various primer sets under uniform experimental conditions to improve accuracy of lymphoma diagnostic and provides a recommendation for achieving the highest diagnostic precision in lymphoma clonality analysis.

## 1 Introduction

Cats are popular companion animals all over the world. Among the malignancies in cats, lymphomas are one of the most important (1). Most lymphomas arise from the two main lineages of lymphocytes, the B and T lymphocytes, whereby many different subtypes with different biological behavior exist (2). Both chronic inflammation and lymphoma cause lymphocyte proliferation. The two entities can be so similar histologically and cytologically that differentiation can be extremely difficult. A classic example of this diagnostic dilemma is the often impossible differentiation of chronic enteritis from alimentary lymphoma (3). While the cells of a lymphoma and an inflammation can look very similar morphologically, there are significant and diagnostically helpful differences at the genetic level. The cells of a lymphoma are usually derived from a single transformed cell and are genetically uniform (clonal) in their antigen receptor genes, whereas the cells of an inflammation are recruited from different cells and therefore differ in their antigen receptor genes (polyclonal) (4–6). Assessment of this clonality by PCR has been shown to aid in precise diagnosis finding (7, 8). Antigen receptor genes are rearranged during lymphocyte development so that each lymphocyte contains a unique gene sequence in that region of the genome (9). T cells directly arrange the variable (V) genes of the T cell receptor gamma (TRG) gene with the connecting (J) genes in multiple cassettes (10–12). In immunoglobulin (IG) light chains, i.e., IG kappa (IGK) and IG lambda (IGL), the V genes are also directly rearranged with the J genes, whereas in IG heavy chains (IGH), the diversity (D) and J genes are rearranged in a first step. The rearranged D-J genes are then fused to a V gene (9). For immunoglobulin light chains, the IGK genes are rearranged first. If this does not result in a functional protein, rearrangement of the IGL genes is initiated and the IGK allele is inactivated by rearrangement of the so-called kappa deleting element (Kde) (10, 13, 14). Because rearrangements involve the random insertion and deletion of nucleotides, rearranged genes differ in length between different lymphocytes. This principle can be applied in PCR assays to distinguish lymphocyte clones based on gene size. Comparison of antigen receptor genes from different lymphocytes reveals regions with varying degrees of variability within these genes. The framework regions (FR) show low variability between different lymphocytes, whereas the complementarity determining regions (CDR) show high variability. Here, CDR3, which covers the junctional regions between rearranged genes, exhibits the highest degree of variability (15). Amplification of CDR3 with flanking primers in the framework regions and subsequent separation of the products by length reveal a uniform product in a clonal neoplastic population and a range of different products in reactive polyclonal populations of lymphocytes (7, 8).

Several primer sets have been developed to assess clonality in cats (6, 10, 16–21). In T-cell lymphomas, TRG genes are the preferred target. This is the first locus to be rearranged and is therefore rearranged not only in gamma-delta T cells but also in alpha-beta T cells (22, 23). The feline TRG locus contains 12 TRGV genes (including pseudogenes) belonging to 6 subsets, 12 TRGJ genes (including pseudogenes and open reading frames), and 6 TRGC genes (including pseudogenes) arranged in 5 complete and 1 incomplete V-J-(J)-C cassettes (24). In B-cell lymphomas, most primer sets target immunoglobulin heavy chain (IGH) genes. There are three subsets of IGHV genes in cats (25). The IGHV3 subgroup is most likely to be involved in rearrangements (18–20), whereas the IGHV1 subgroup is less likely to be involved in rearrangements (18, 19). The IGHJ genes can be divided into two frequently rearranged variants and several less frequent variants (18–20). Rout et al. expanded the range of potential targets for assessing clonality in B-cell populations in cats by adding an assay that additionally includes incomplete rearrangement of the IGH genes (IGH-DJ rearrangement), rearrangement of the IGL genes, and rearrangement of the Kde genes (10). In 2021, an additional assay for clonality analysis of feline T-cell lymphomas was published that incorporated T-cell receptor beta, delta, and gamma loci (21).

Direct comparison of primer sets for analyzing clonality in cats is challenging due to differences in methodological approaches, sample sets, and laboratory conditions used in their original published experimental design (Table 1). Early studies used polyacrylamide gel electrophoresis (PAGE) to separate PCR products, whereas more recent studies separate products by capillary electrophoresis, which is currently considered the gold standard for clonality analysis. Some published primer sets do not cover all the gene subgroups described, and the method of fixation also varies (fresh tissue or cells vs. FFPE samples).

**Table 1:** Comparison of published primer sets currently used for clonality analysis of feline lymphomas.

	Authors	Target(s)	No. of cases	Material	Detection	Performance
T cells	Moore et al. (6)	TRG	28 neoplastic 12 reactive	FFPE	PAGE	Sen: 78% (CI: 59–92%) Spe: 100% (CI: 74–100%)
	Weiss et al. (16)	TRG	19 neoplastic 10 reactive	FFPE	PAGE	Sen: 68% (CI: 43–87%) Spe: 100% (CI: 3–100%)
	Mochizuki et al. (17)	TRG	30 neoplastic 34 reactive	FFPE, Fresh	CE	Sen: 87% (CI: 69–96%) Spe: 97% (CI: 85–100%)
	Rout et al. (10)	TRG	30 neoplastic 11 reactive	Fresh	CE	Sen: 97% (CI: 83–100%) Spe: 100% (CI: 72–100%)
B cells	Werner et al. (20)	IGH	22 neoplastic 4 reactive	FFPE	PAGE	Sen: 68% (CI: 45–86%) Spe: 100% (CI: 40–100%)
	Henrich et al. (19)	IGH	10 neoplastic 10 reactive	FFPE	PAGE	Sen: 70% (CI: 35–93%) Spe: 100% (CI: 69–100%)
	Mochizuki et al. (18)	IGH	20 neoplastic 2 reactive	FFPE, Fresh	CE	Sen: 85% (CI: 65–96%) Spe: 100% (CI: 3–100%)
	Rout et al. (10)	IGH (compl.) IGH (incompl.) Kde IGL	38 neoplastic 11 reactive	Fresh	CE	Sen: 87% (CI: 72–96%) Spe: 100% (CI: 72–100%)

TRG, T-cell receptor gamma genes; FFPE, formalin-fixed and paraffin-embedded; PAGE, polyacrylamide gel electrophoresis; Sen, sensitivity; Spe, specificity; CI, 95% confidence interval; CE, capillary electrophoresis; IGH, immunoglobulin heavy chain genes; Kde, kappa deleting element; IGL, immunoglobulin light chain genes.

Therefore, it is important to conduct a systematically and controlled comparison. Here we present a comparative analysis of PCR primer sets for clonality detection in feline lymphomas. Different primer sets commonly used in diagnostics to date were compared to best assess clonality in feline lymphomas under standardized laboratory and sample conditions. We identified combinations of primer sets for T-cell lymphoma and B-cell lymphoma clonality diagnostics with a significantly higher diagnostic accuracy than the individual primer sets used to date.

## 2 Materials and methods

### 2.1 Comparison of the primer sets by alignment

For the primer sets binding at the same targets, the sequences of the primers from the different assays were aligned with the corresponding sequences of the feline antigen receptor genes to evaluate and visualize the spatial relationship of the primers. Alignment was performed using BioEdit version 7.2.0 (26), and the sequences of the feline antigen receptor genes were taken from previous studies (19, 27).

### 2.2 Patient samples

FFPE samples from 31 cats with T-cell lymphomas, 29 cats with B-cell lymphomas (Table 2), and 11 cats with reactive lymphoid proliferations (Table 3) were included in the study. The diagnosis of lymphoma and reactive lymphoid lesions was made by histology [according to WHO criteria (2)] and immunohistochemistry against the T-cell marker CD3 (polyclonal rabbit anti-human CD3, Dako, Hamburg, Germany) and the marker CD45R (rat anti-mouse CD45R, clone B220/RA3-6B2 [Ly 5], Cedarlane, Burlington, Ontario, Canada) to identify B cells (28–31) according to routine protocols (32). All cases were reviewed by two senior pathologists (KK, MH), and only cases with an unequivocal diagnosis were included.

**Table 2:** Patient characteristics, diagnosis, and tumor location of cats with lymphomas.

Case	Breed	Age (years)	Gender	Diagnosis	Tumor location
**T-cell lymphomas**					
1	N/A	4	F	Intestinal T-cell lymphoma, EATL type 1	Intestine
2	ESH	9	FS	Mycosis fungoides	Skin
3	ESH	13	FS	Intestinal T-cell large granular lymphocyte lymphoma (LGL)	Intestine
4	ESH	N/A	MC	Intestinal T-cell lymphoma, EATL type 1	Intestine
5	ESH	10	MC	Intestinal T-cell lymphoma, EATL type 1	Intestine
6	ESH	8	MC	Mycosis fungoides	Skin
7	ESH	14	F	Intestinal T-cell lymphoma, EATL type 1	Intestine
8	ESH	14	F	Mycosis fungoides	Skin
9	Turkish Angora	12	FS	Intestinal T-cell lymphoma, EATL type 2	Intestine
10	ESH	12	MC	Intestinal T-cell lymphoma, EATL type 1	Intestine
11	ESH	11	MC	Extranodal peripheral T-cell lymphoma NOS	Gingiva
12	ESH	12	FS	Mycosis fungoides	Skin
13	Persian	13	FS	Cutaneous peripheral T-cell lymphoma NOS	Skin
14	ESH	7	MC	Extranodal peripheral T-cell lymphoma NOS (FeLV-positive)	Pericardium
15	N/A	4	F	Extranodal peripheral T-cell lymphoma NOS	Mediastinum
16	ESH	14	FS	Mycosis fungoides	Skin
17	ESH	16	FS	Extranodal peripheral T-cell lymphoma NOS	Urinary bladder
18	ESH	14	F	Extranodal peripheral T-cell lymphoma NOS	Liver
19	ESH	1	FS	Extranodal peripheral T-cell lymphoma NOS	Mediastinum
20	ESH	12	FS	Cutaneous peripheral T-cell lymphoma NOS	Skin
21	ESH	10	F	Nodal peripheral T-cell lymphoma NOS	Lymph node
22	N/A	10	FS	Intestinal T-cell lymphoma, EATL type 2	Intestine
23	Norwegian Forrest Cat	12	MC	Extranodal peripheral T-cell lymphoma NOS	Liver
24	N/A	9	F	Intestinal T-cell lymphoma, EATL type 1	Liver
25	Maine Coon	16	FS	Cutaneous peripheral T-cell lymphoma NOS	Skin
26	ESH	17	FS	Intestinal T-cell lymphoma, EATL type 1	Intestine
27	ESH	12	FS	Intestinal T-cell lymphoma, EATL type 1	Intestine
28	ESH	N/A	FS	Intestinal T-cell lymphoma, EATL type 1	Intestine
29	N/A	4	MC	Extranodal peripheral T-cell lymphoma NOS	Eye
30	Maine Coon	11	MC	Extranodal peripheral T-cell lymphoma NOS	Omentum
31	ESH	14	MC	Intestinal T-cell lymphoma, EATL type 2	Intestine
**B-cell lymphomas**					
32	ESH	6	MC	Diffuse large B-cell lymphoma	Nasal cavity
33	ESH	3	MC	Diffuse large B-cell lymphoma	Intestine
34	N/A	2	MC	T-cell-rich large B-cell lymphoma	Intestine
35	ESH	8	FS	Follicular lymphoma grade III	Lymph node
36	Siam	9	FS	Diffuse large B-cell lymphoma	Intestine
37	ESH	N/A	F	Follicular lymphoma grade III	Lymph node
38	ESH	11	FS	Diffuse large B-cell lymphoma	Brain
39	ESH	11	F	Follicular lymphoma grade III	Lymph node
40	ESH	5	MC	Diffuse large B-cell lymphoma	Tonsil
41	ESH	14	MC	Diffuse large B-cell lymphoma	Lymph node
42	Siamese	15	MC	Diffuse large B-cell lymphoma	Spleen
43	Maine Coon	13	FS	Diffuse large B-cell lymphoma	Intestine
44	ESH	2	MC	Follicular lymphoma grade III	Lymph node
45	N/A	N/A	F	T-cell-rich large B-cell lymphoma	Lymph node
46	ESH	11	FS	Diffuse large B-cell lymphoma	Kidney
47	ESH	5	M	Plasmablastic lymphoma	Mesenterium
48	ESH	16	MC	Diffuse large B-cell lymphoma	Olfactory bulb
49	ESH	12	M	B-cell small lymphocytic lymphoma	Omentum
50	ESH	14	FS	T-cell-rich large B-cell lymphoma	Lymph node
51	Maine Coon	11	MC	Diffuse large B-cell lymphoma	Stomach
52	ESH	N/A	FS	MALT lymphoma	Intestine
53	ESH	9	MC	Diffuse large B-cell lymphoma	Intestine
54	N/A	N/A	N/A	Diffuse large B-cell lymphoma	Intestine
55	N/A	15	FS	Diffuse large B-cell lymphoma	Intestine, Mesentery
56	BSH	12	MC	Diffuse large B-cell lymphoma	Intestine
57	Siamese	9	FS	Diffuse large B-cell lymphoma	Lung
58	ESH	10	FS	Follicular lymphoma grade III	Mesentery
59	N/A	14	M	B-cell small lymphocytic lymphoma	Pancreas
60	ESH	11	FS	Diffuse large B-cell lymphoma	Skin

N/A, not available; ESH, European short hair; BSH, British short hair; F, female; M, male; S, spayed; C, castrated; EATL, enteropathy-associated intestinal T-cell lymphoma; NOS, not otherwise specified.

**Table 3:** Patient characteristics, diagnosis, and tumor location of cats with reactive lymphocytic proliferations.

Case	Breed	Age (years)	Gender	Diagnosis	Site sampled
61	N/A	N/A	MC	Follicular hyperplasia	Lymph node
62	Maine Coon	1	M	Follicular hyperplasia	Lymph node
63	BSH	4	MC	Follicular hyperplasia	Lymph node
64	Scottish Fold	1	F	Follicular hyperplasia	Lymph node
65	N/A	6	MC	Follicular hyperplasia	Lymph node
66	ESH	6	M	Follicular hyperplasia	Lymph node
67	ESH	13	FS	Follicular hyperplasia	Lymph node
68	ESH	2	FS	Granulomatous lymphadenitis	Lymph node
69	ESH	11	FS	Chronic enteritis	Small intestine
70	N/A	5	MC	Follicular hyperplasia	Spleen
71	BSH	0.83	MC	Petechia	Thymus

N/A, not available; ESH, European short hair; BSH, British short hair; F, female; M, male; S, spayed; C, castrated.

### 2.3 Clonal controls

For TRG rearrangements, DNA from a T-cell line (S87) with known TRG rearrangement (33) was used. For IGH rearrangements, DNA from a diffuse large B-cell lymphoma with confirmed clonal electrophoresis pattern (19) served as clonal control. For primer sets that failed to amplify this lymphoma, plasmids containing the corresponding variants of the *IGHV* genes and the *IGHJ* genes were used (19).

### 2.4 DNA extraction and quality

DNA from FFPE samples was isolated from paraffin blocks using the QIAamp DNA FFPE Tissue Kit^®^ (QIAGEN GmbH, Hilden, Germany) according to the manufacturer’s instructions with the following modifications: In cases with small areas of tumor cells, material from the region of interest was manually scraped from the blocks using a sterile microtome blade to minimize the amount of DNA from non-neoplastic cells. DNA from cell culture material (cell line S87) was isolated according to a modified protocol of Miller et al. (34). Briefly, 1 mL of cell suspension (1 × 10^6^ cells/ml) was centrifuged (15.000 × *g*, 5 s). The cell pellet was incubated with 300 μL cell lysis buffer (10 mM Tris–HCl, 400 mM NaCl and 2 mM Na_2_EDTA, pH 8.2) at 37°C until homogeneous. RNA was removed by adding RNase A and incubating at 37°C for 30 min. After cooling to room temperature, the protein in the solution was precipitated by adding 100 μL protein precipitation solution (saturated NaCl, approximately 6 M) and pelleted after vortexing vigorously. The DNA in the supernatant was precipitated in 300 μL isopropanol and after vortexing and centrifugation, the pellet was washed in 300 μL 70% ethanol. The DNA was resuspended in DNA hydration solution (10 mM Tris–HCl, 0.2 mM Na_2_EDTA, pH 7.5). All chemicals and enzymes were purchased from Carl Roth GmbH & Co. KG, Karlsruhe, Germany. DNA concentration was determined photometrically (NanoDrop 2000; Thermo Scientific, Waltham, MA, USA) after homogenization at 63°C. All samples were stored at −20°C. DNA quality was assessed by amplifying fragments of approximately 300 bp (19), approximately 188 bp (18), and approximately 150 bp (19) from the non-rearranged portions of the feline genome.

### 2.5 PCR protocols for the analysis of IG and TRG rearrangements

For each case, all primer sets used (Supplementary Table 1) were tested on the same DNA isolation to avoid bias due to variability in isolated DNA. To facilitate detection of pseudoclonality, all reactions were run in duplicate (T-cell lymphomas) or triplicate (B-cell lymphomas) at 50 ng DNA per 25 μL PCR reaction, as recommended by Keller et al. (7). DNA was amplified using Bioline MyTaq^™^ HS Mix (Bioline GmbH, Luckenwalde, Germany) and the respective primers (purchased from Biomers.net GmbH, Ulm, Germany). Primers were used in the indicated combinations and concentrations (Supplementary Table 1). For use in capillary electrophoresis, the primer sets were labeled with fluorescent dyes so that each amplicon carried a fluorescent dye molecule (Supplementary Table 1). Cycling conditions were as indicated for the primer sets (Supplementary Table 2) with the following modifications: If not included in the original cycling conditions, an initial activation step (5 min, 95°C) of Bioline MyTaq^™^ HS (hot start) polymerase was added. In addition, a terminal elongation step (10 min, 72°C) was added to ensure 3′-adenine overhangs and thereby minimizing “plus A” artifacts in capillary electrophoresis. In the protocols of Mochizuki et al. (17, 18), the terminal elongation step was already included. A non-template control was used for all reactions.

### 2.6 Capillary electrophoresis

Amplicons were separated on an ABI Prism® 310 Genetic Analyzer (Applied Biosystems® by life technologies, Darmstadt, Germany). PCR products were analyzed either undiluted or diluted in water after PCR. The standard dilution was either 1:5 or 1:10 (depending on the primer set). The (diluted) PCR product was denatured in formamide (Hi-Di™ Formamide, Applied Biosystems® by life technologies, Darmstadt, Germany) mixed with LIZ size standard (GeneScan™ 500 LIZ™ dye Size Standard, Applied Biosystems® by life technologies, Darmstadt, Germany) at 95°C for 5 min, followed by 10 min incubation on ice. Injection was performed at 15,000 V for 5 s. Electrophoresis was performed at 60°C, 15,000 V and 10 mA laser current. If the electrophoresis result was inadequate, the dilution and/or injection time were adjusted: If the signals reached the detection maximum, the dilution was increased. If the peaks were too small or absent, the injection time was increased to 10 s. If this did not improve the results, electrophoresis was repeated with less diluted or undiluted PCR product.

### 2.7 Analysis of results

Graphical representation and size analysis were performed using Peak Scanner™ software version 1.0 (Applied Biosystems® by life technologies, Darmstadt, Germany). Microsoft Excel 2019 was used for statistical analysis of the data. Electrophoresis results were interpreted/described according to published guidelines (7, 8). To objectify the comparison of the different primer sets, some criteria were defined as follows:

According to the commonly used definition of a reference interval, the **expected size range** for each primer set was defined as the central 95% of the reference distribution (size distribution of all amplicons of the polyclonal controls of the respective set). Therefore, the expected size range was calculated as μ ± 2σ of the size of the amplicons of the polyclonal controls (with μ = mean and σ = standard deviation).

**Specific peaks** were defined as peaks within the expected size range [± 10 bp, according to Rothberg et al. (17)]. All peaks outside the expected size range were considered **non-specific peaks**. The latter category also included peaks that were reproducible in unrelated samples and/or non-template controls (so-called “constant peaks”), regardless of whether these peaks occurred within or outside the expected size range (7, 8).

**Reproducible peaks** were considered as such if peaks of the same size (in bases) occurred in duplicate/triplicate reactions. Because the analytical software calculates peak size based on the added size standard, the size of a peak is rounded to two decimal places. Peaks were considered equal in size if the peak size did not differ from the mean of the two peak sizes by more than ±0.49 bases.

**Multiple peaks** have been defined as ≥3 peaks (8). In this study, multiple peaks were defined as 3 to 5 distinct peaks without background (6, 20). Signals of less than 50 relative fluorescence units (RFU) were considered **background noise**, regardless of whether they were “peak-like” structures that stood out from the surrounding background fluorescence signal.

**Small peaks/low peak height** were defined as peaks between 50 RFU and the “limit of quantification” (LOQ) of the ABI Prism® 310 Genetic Analyzer. The LOQ is the lower threshold of the interval of fluorescence signal strength with a linear relationship between signal strength and number of DNA fragments. For the ABI Prism® 310 Genetic Analyzer the LOQ is 150 RFU.

All electrophoresis patterns were assigned to one of the following categories:

**No specific peaks** were defined as the absence of specific peaks as defined above. If DNA quality was adequate, the sample was interpreted as not amplifiable with the according primer set. If DNA quality was poor, the absence of specific peaks could be due to a non-binding primer set and/or a generally non-amplifiable DNA. Therefore, these samples were classified as non-evaluable.

**Figure 1:**
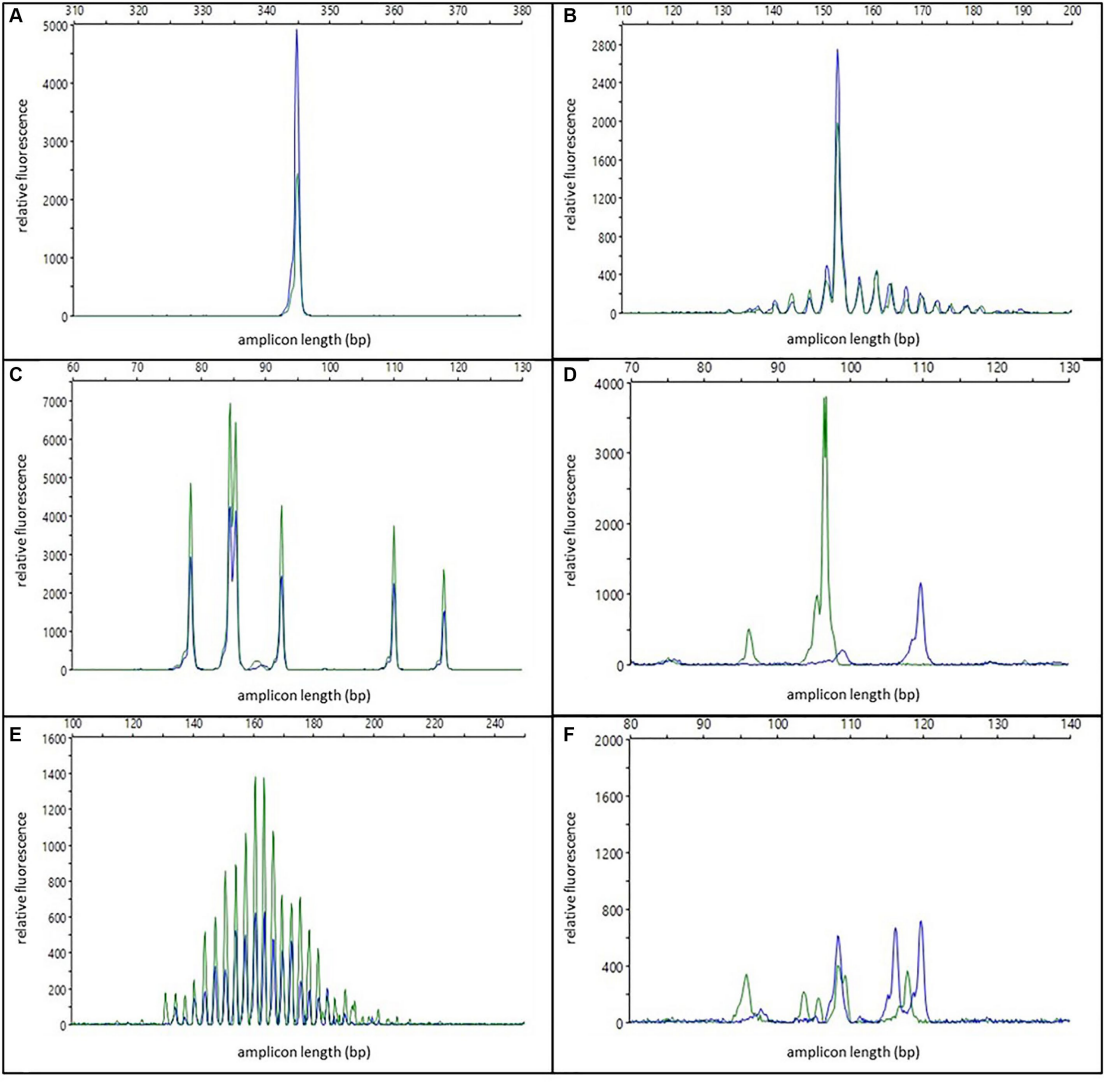
Examples of different electrophoresis patterns; blue and green colors represent duplicate reactions; **(A)** one specific reproducible peak/clonal; **(B)** one specific reproducible peak with background/clonal with polyclonal background; **(C)** multiple specific reproducible peaks/oligoclonal; **(D)** two non-reproducible peaks/pseudoclonal; **(E)** Gaussian curve/polyclonal; **(F)** multiple specific non-reproducible peaks/irregularly polyclonal.

**One or two specific, reproducible peaks** were considered indicative of a clonal population (see Figure 1A). In cases with two peaks, either a biclonal population or a biallelic rearrangement was assumed. For the interpretation of clonality, reproducibility was mandatory for at least one of the peaks. Peaks in this category with low peak height (50 to 150 RFU) were interpreted with caution and only as indicative of a clonal population. The modifier “**with background**” was applied when the reproducible peaks were accompanied by a pattern indicative of a polyclonal/oligoclonal population in the same tissue (see Figure 1B). To distinguish clonal peaks from background, clonal peaks in all duplicate/triplicate reactions had to be at least twice as high as adjacent peaks (2:1 ratio). This corresponds to 5% clonal cells in the sample (35). Samples that had reproducible peaks with a background below the 2:1 ratio were interpreted as “polyclonal with minor clones of uncertain significance.”

**Multiple specific, reproducible peaks (≤5)** were interpreted as the typical pattern of an oligoclonal population (see Figure 1C). In contrast to other publications (10), these cases were regarded as non-clonal and therefore excluded from the sensitivity calculation.

**One or two non-reproducible peaks** were considered as pseudoclonal results due to random amplification of different single clones of an oligoclonal or polyclonal population in repeated PCR reactions of samples with poor DNA quality (see Figure 1D).

Electrophoresis patterns with multiple (>5) peaks arranged in a more or less “bell-shaped” **Gaussian curve**, were interpreted as polyclonal populations (see Figure 1E). This interpretation could be modified: Samples with either irregularly shaped curves, samples with regular curves in one reaction but only three to five single peaks in the replicated reactions, or samples with non-reproducible discernable peaks that did not have a 2:1 ratio to the adjacent peaks in the replicated reaction were designated as irregularly polyclonal. Gaussian curves with low height (50 to 150 RFU) were interpreted as “weakly polyclonal.”

**Multiple specific, non-reproducible peaks** were also interpreted as irregularly polyclonal (see Figure 1F).

Samples that did not fit into one of the aforementioned categories, e.g., samples with peaks only within background noise, samples with no specific peaks in one reaction and at least three peaks or a Gaussian curve in the replicates, or samples with one or two specific peaks without background in one sample, but a Gaussian curve or multiple non-reproducible peaks in the replicates, were regarded as **non-evaluable**.

## 3 Results

### 3.1 Comparison of primer sets by alignment

**Figure 2:**
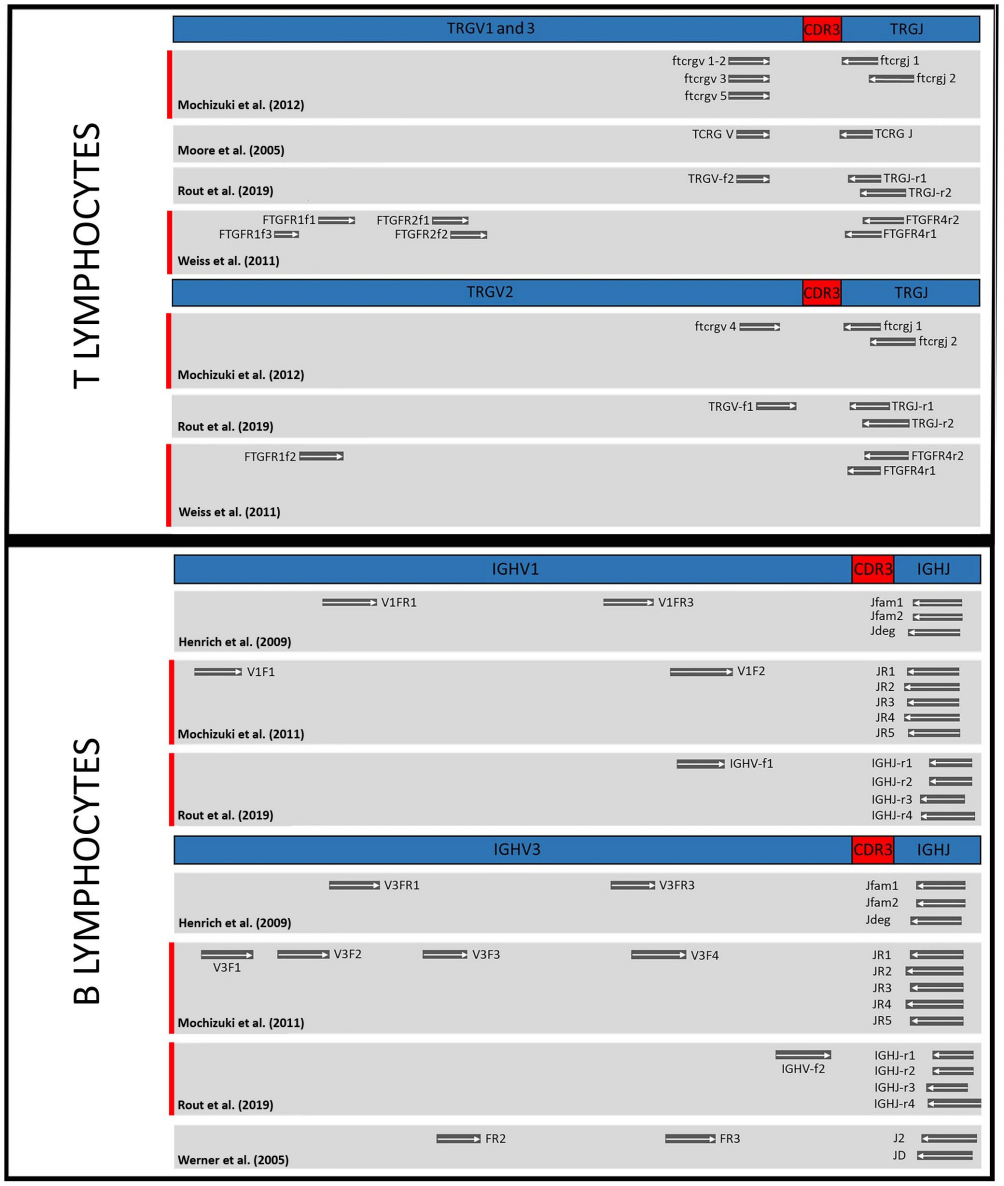
Comparison of primer binding sites of the different test sets in the genes of T cell receptor gamma (TRG) and immunoglobulin heavy chain (IGH). TRGV1, 2 and 3, subgroups of T cell receptor gamma variable region genes; TRGJ, T cell receptor gamma joining region genes; CDR3, complementarity determining region; IGHV1 and 3, immunoglobulin heavy chain variable region subgroups; IGHJ, immunoglobulin heavy chain joining region genes. Highlighted in red: Primer combinations that together correctly classified the most populations as clonal or polyclonal in the study.

The spatial relationship of primer sets targeting TRG genes and IGH genes, respectively, is shown in Figure 2. Although there is a clear difference in the sets for individual targets, there is also significant overlap.

### 3.2 Experimental comparison of primer sets

The comparison of the results and performances of the different primer sets is shown in Table 4. In addition to the key figures of the individual primer sets, the corresponding values of the combination of primer sets that together achieved the best test results are also listed (for detailed results of the individual samples see Supplementary Tables 3, 4).

**Table 4:** Results of the comparison of the analyzed primer sets.

Authors		Clonality detected		Performance
		Lymphoma	Polyclonal control	
T cells	Moore et al. (6)	15/31	1/11	Sen: 48% (CI: 30.2–66.9%) Spe: 91% (CI: 58.7–99.8%)
	Weiss et al. (16)	18/31	1/11	Sen: 58% (CI: 39.1–75.5%) Spe: 91% (CI: 58.7–99.8%)
	Mochizuki et al. (17)	23/31	1/11	Sen: 74% (CI: 55.4–88.2%) Spe: 91% (CI: 58.7–99.8%)
	Rout et al. (10)	21/31	0/11	Sen: 68% (CI: 48.6–83.3%) Spe: 100% (CI: 71.5–100%)
	Mochizuki et al. (17) + Weiss et al. (16)	25/31	1/11	Sen: 81% (CI: 61.5–99.8%) Spe: 91% (CI: 58.7–99.8%)
B cells	Werner et al. (20)	9/29	0/11	Sen: 31% (CI: 15.3–50.8%) Spe: 100% (CI: 71.5–100%)
	Henrich et al. (19)	12/29	0/11	Sen: 41% (CI: 23.5–61.1%) Spe: 100% (CI: 71.5–100%)
	Mochizuki et al. (18)	18/29	2/11	Sen: 62% (CI: 42.3–79.3%) Spe: 82% (CI: 48.2–97.7%)
	Rout et al. (10) Exclusion of IGKDE	21/29 17/29	6/11 2/11	Sen: 72% (CI: 52.8–87.3%) Spe: 45% (CI: 10.93–69.21%) Sen: 59% (CI: 38.9–76.5%) Spe: 82% (CI: 48.2–97.7%)
	Mochizuki et al. (18) + Rout et al. (10) Exclusion of IGKDE	21/29	4/11	Sen: 72% (CI: 52.8–87.3%) Spe: 64% (CI: 30.79–89.1%)

Sen, sensitivity; Spe, specificity; CI, 95% confidence interval.

For **T cells**, the best results were obtained by combining the primer sets of Mochizuki et al. (17) and Weiss et al. (16). The primers of Weiss et al. (16), successfully detected clonality in two samples where the primers of Mochizuki et al. (17) indicated polyclonality. Conversely, the primers of Mochizuki et al. (17) detected clonality in seven samples, whereas the primers of Weiss et al. (16) showed polyclonality, no rearrangement or non-evaluable results there. The combination of the sets of Mochizuki et al. (17) and Weiss et al. (16) included all clonal results obtained with the primer sets of Moore et al. (6) and Rout et al. (10), each of which yielded fewer clonal results.

Weiss et al. (16) concluded in their study that by using the reverse primers FTGFR4r2 and FTGFR4r3 clonality could only be detected in 58% of lymphomas and false clonal results occurred in 86% of the polyclonal control group. Therefore, they did not recommend the use of these primers. However, in the present study, the sensitivity of PCR reactions with FTGFR4r2 was 45% and the specificity 91%. The clonal result of control sample 69 as cause of the lower specificity could also be reproduced with several other primer sets. Moreover, in four samples, clonality was detectable only with FTGFR4r2. Therefore, the results of the present study suggest that the use of this primer may be valuable.

The primer set of Moore et al. (6), covering only TRGV subgroup 1 genes, showed clonality in 15/31 lymphomas, suggesting that TRGV subgroup 1 was most frequently rearranged in this study. In 6/31 samples detection of clonality was successful with the primer sets of Weiss et al. (16) and/or Mochizuki et al. (17) (covering all three TRGV subgroups) and additionally with the set of Rout et al. (10) (covering TRGV subgroups 1 and 2), but not with that of Moore et al. (6). This distribution suggests that these neoplastic clones utilized TRGV subgroup 2 genes. In 4/31 samples, clonality was detected with the primer sets of Weiss et al. (16) and/or Mochizuki et al. (17), but not with those of Moore et al. (6) and Rout et al. (10). This indicates the use of TRGV subgroup 3 genes.

In six T-cell lymphomas, clonality could not be demonstrated with any system. All primer sets except that of Rout et al. (10) showed clonality in one polyclonal control sample (no. 69, chronic enteritis). However, with the exception of the FR2f1 PCR by Weiss et al. (16), a background polyclonal population was detectable in every PCR. The primer combinations with which clonality could not be detected gave clear polyclonal results. A possible explanation for this apparent false positive clonality detection could be a limited number of lymphocytes in the small intestinal biopsy. On the other hand, progression of chronic enteritis to intestinal lymphoma has been reported (6, 36). Therefore, it cannot be excluded that this sample actually contains a neoplastic population of lymphocytes.

For **B cells**, the best results were obtained by combining the primer sets of Mochizuki et al. (18) and Rout et al. (10), excluding the PCR for rearrangement of the Kde genes (IGKDE-PCR). IGKDE-PCR achieved a sensitivity of 38%. However, six clonal results were detected in the polyclonal control group, resulting in a specificity of only 45%. This was due to the occurrence of reproducible peaks at around 189 and 193 bp (see Table 5). Most samples in the lymphoma group assessed as clonal also showed these peaks. It is unclear whether these are specific PCR products or nonspecific “constant peaks” that need to be excluded from the evaluation. Therefore, this set was not included in further calculations. However, with the combination of the primer sets of Mochizuki et al. (18) and Rout et al. (10) modified in this way, clonality was detected in three samples with only one of these two sets.

**Table 5:** Constant peaks.

**Werner et al. (20)**	
FR3/JD PCR	Approx. 128 bp
**Rout et al. (10)**	
IGHV-f1 PCR	Approx. 149 bp and 154 bp
IGH-DJ PCR	Approx. 77 and 85 bp
IGKDE PCR	Approx. 189 and 193 bp
TRGJ-r1 PCR	Approx. 73 bp and 77 to 79 bp

All clonal results obtained with the primer set of Werner et al. (20) and Henrich et al. (19) were also obtained by the set of Mochizuki et al. (18).

Sample 55 was the only B-cell lymphoma in which detection of clonality with IGHV1-specific primers was successful, supporting the hypothesis that rearrangement of this subgroup is rare in the cat population as a whole (18, 19, 37).

In eight B-cell lymphomas, clonality detection was not possible.

Four polyclonal control samples showed a clonal result. Sample 71 was thymic tissue and sample 69 was chronic small intestinal enteritis with a possible low number of B cells in the samples. Samples 65 and 67 showed a polyclonal population in addition to the clonal peaks. These peaks had fragment lengths in the middle of the Gaussian curve, i.e., in the fragment length range where one would statistically expect the most frequent rearrangements. Thus, these peaks could represent multiple clones of the same length that cannot be distinguished by capillary electrophoresis.

In addition to the results of the primer comparison, additional insights were gained with regard to the technical performance of the assay.

The **DNA quality** of all FFPE samples was sufficient for amplification of a 150 bp long fragment. Amplification of the 300 bp long fragment failed in one polyclonal control sample, six B-cell lymphomas and six T-cell lymphomas. However, the 188 bp fragment could be amplified in all these samples. As proposed by Lenze (38), DNA purity was checked photometrically (Supplementary Table 5). Suboptimal A260/A280 and A260/A230 ratios were present in 28% of B-cell lymphomas (8/29) and 35% of T-cell lymphomas (11/31), respectively. However, the effect on the sensitivity of the assay appeared to be negligible, as detection of clonality failed in 20% of samples with suboptimal purity and in 27% of samples with optimal ratios. With the exception of one sample with suboptimal purity, amplicons of at least 188 bp could be generated in all samples.

When performing capillary electrophoresis, it is important to keep the **signal strength** (peak height in RFU) in the evaluable range, i.e., in the range with a linear relationship between the number of DNA fragments of a certain length and the signal strength (39). For many samples, this was not the case when using the undiluted PCR product. The peak height exceeded the so-called “limit of linearity” and the electrophoresis images were not interpretable. In these cases, **predilution of the PCR product** was required. Neither the concentration of the isolated DNA nor the purity of the DNA allowed prediction of the dilution required. However, clonal samples generally required a higher dilution. The only publication on PCR clonality diagnostics by capillary electrophoresis that mentions the necessity of predilution (10) specifies a standard tenfold dilution for each sample. However, in our study, a dilution of up to 1:100 was required when using the same test system. There seems to be a correlation with the efficiency of the amplification and most likely also with the technical parameters of the analytical instrument. Therefore, an examination of the optimal dilution for each primer set might be necessary, which would have to be additionally adjusted depending on the evaluability of the electrophoresis results for individual samples.

For most assays, the calculation of the **expected size range** of the primer sets resulted in larger ranges than in the respective original publications (Supplementary Table 6).

In some PCR reactions, **reproducible amplicons** were generated that were more than 10 bp outside the expected size range. Sequence analysis is required to determine whether these are specific rearrangements (40).

In addition, **constant peaks** were detected in some PCR reactions (see Table 5).

## 4 Discussion

In recent years, a variety of diagnostic assays with multiple primer sets has been developed to assess clonality in feline lymphomas. Each primer set has been validated in the original publications and subsequent studies. However, accurate comparison of these primer sets is complicated by the different approaches and material used. In this study, we employed a standardized approach to compare various primer sets, ultimately offering a conclusive recommendation for achieving high diagnostic precision in feline lymphoma clonality analysis.

In T-cell lymphomas, most populations could be correctly assigned as clonal or polyclonal by combining the primer sets of Weiss et al. (16) and Mochizuki et al. (17). This is not surprising, as those two can detect a broad range of possible genetic variants, including targets in all framework regions. However, the use of the primer set of Weiss et al. (16) is relatively time- and material-consuming due to the numerous PCR reactions. For use in routine diagnostics, particularly on FFPE tissues, considerations should therefore be given to use the primers of Mochizuki et al. (17) as a first step, which also have the advantage of shorter amplicons. If clonality cannot be demonstrated and lymphoma is still strongly suspected, the primer set of Weiss et al. (16) could be used subsequently.

In six T-cell lymphomas clonality could not be detected with any system. The lack of specific amplification products may have numerous causes other than poor DNA quality, which has a negative impact especially in systems with longer fragment lengths. Insufficient coverage of different genetic variants by the available primers, mutation of primer binding sites, which however plays a role particularly in B lymphocytes due to somatic hypermutation, and chromosome aberrations are possible causes (7). According to Wang et al. (41), the absence of target amplification combined with good amplification of control genes may also indicate a lack of lymphocyte DNA in the sample. Finally, in the absence of evidence of clonality by all primer sets, the histologic diagnosis must also be self-critically questioned. Although the cases were classified as lymphomas according to WHO histologic criteria (2) and by immunohistochemical determination of the immunophenotype of the lymphocytes, it cannot be completely excluded that they are in fact reactive, i.e., non-neoplastic lesions.

For B-cell lymphomas, the best result was obtained by combining the sets of Mochizuki et al. (18) and Rout et al. (10), but without using IGKDE-PCR. Even without the analysis of Kde rearrangements, the combination of these two primer sets covers the broadest range of genetic variants, which explains the good overall result. Kde rearrangement analysis was excluded due to the low specificity of 45% based on the constant peaks in the control group. It is conceivable that generally low length variability at this locus leads to the formation of PCR products of similar length, but with different sequence. Capillary electrophoresis could then fail to distinguish these products.

In eight B-cell lymphomas, no clonality detection was possible. As mentioned above, the phenomenon of somatic hypermutation in B-cell lymphomas, probably has a major impact on the integrity of the primer binding sites especially in FR3. This can also be assumed as the cause of the lower sensitivity of the B-cell clonality assays compared with the T-cell clonality assays in the presented study (18, 20).

Overall, the present work did not detect any influence of lymphoma type on the sensitivity of certain primer sets or preferential rearrangement of certain targets. However, the number of each lymphoma type in the sample material was low. In addition, there was a clear disparity in the frequency of the different types: approximately 42% of the T-cell lymphomas were intestinal forms (13/31) and approximately 59% of the B-cell lymphomas were diffuse large B-cell lymphomas (DLBCLs) (17/29).

So-called “constant peaks” (see Table 3), i.e., peaks that are reproducible in many or even all samples, can have different causes (7). Non-specific amplification occurs mainly when only a few specific primer binding sites are present, i.e., in samples with a low number of B or T lymphocytes. This effect is particularly common in multiplex PCR reactions because of greater competition for template DNA. For clonality tests in human medicine, there are numerous descriptions of such non-specific amplification products, which must be taken into account in the evaluation (8). In veterinary test systems, such descriptions are rare. To avoid misinterpretation, more detailed information on this phenomenon would be useful. Keller et al. (7) mention low CDR3 diversity in certain V-J recombinations in canine T cells. This phenomenon has also been described in humans and is referred to as “canonical rearrangements” (42, 43). This could also explain the occurence of “constant peaks” in feline lymphocyte samples.

The further development of PCR based clonality diagnostics in veterinary medicine in general and in the cat in particular requires a prospective study on a larger scale using sample material relevant to practice, i.e., doubtful cases in which lymphoma is clearly suspected but no definite diagnosis is possible on the basis of preliminary examinations. Analogous to the study situation in dogs (44), a comparison of different sample material (FFPE tissue, fresh tissue, fine needle aspirates, cell pellets) should be performed. To assess the relevance of PCR clonality analysis results for disease progression, response to chemotherapy, and prognosis of different lymphoma types, a detailed clinical follow-up would be required.

The ongoing advances in feline antigen receptor gene analysis need to be incorporated into clonality testing. Subsequent to the completion of our study, Radtanakatikanon et al. presented new findings on the feline T cell receptor using a newly developed assay (21). This includes the T cell receptor delta, beta, and gamma genes, along the multicassette structure of the feline TRG locus. Direct incorporation of this novel primer set into our current comparison would have compromised the strict comparability of the initial samples used and thus undermined the robustness of our study. While this is a limitation of our study, conducting a separate comparative analysis of the new assay alongside the primer sets of Weiss et al. (16) and Mochizuki et al. (17) should allow for an evaluation of the performance of this new assay.

In accordance with Keller et al. (7), adopting standardized performance metrics and evaluation criteria as in human medicine (45) would be beneficial to ensure comparability of test results between laboratories.

In summary, by systematically comparing different primer sets, this study revealed useful combinations of assays [Weiss et al. (16) and Mochizuki et al. (17) for T cells and Mochizuki et al. (18) and Rout et al. (10) for B cells] for clonality analysis of feline lymphomas.

Furthermore, the results provided clarification of some definitions that may be useful for interpreting the results of clonality analysis in feline lymphomas.

## Data availability statement

The raw data supporting the conclusions of this article will be made available by the authors, without undue reservation.

## Ethics statement

Ethical approval was not required for the study involving animals in accordance with the local legislation and institutional requirements because the study was conducted on fixed archive material.

## Author contributions

AW: Methodology, Writing – review & editing, Writing – original draft, Investigation, Funding acquisition, Conceptualization. WH: Investigation, Writing – review & editing, Validation, Methodology. KK: Writing – review & editing, Validation, Investigation. CH: Writing – review & editing, Validation, Supervision. MH: Writing – review & editing, Validation, Supervision, Methodology, Investigation, Conceptualization.
