# Supplementary material for: Comparative analysis of primer sets for the assessment of clonality in feline lymphomas
Source: Front Vet Sci. 2024 May 7;11:1356330. doi: 10.3389/fvets.2024.1356330 (PMC11106357; doi:10.3389/fvets.2024.1356330)
Supplement: Supplementary file 1 [file Table_1.DOCX]

| **Supplementary table 1:** Primer systems for the analysis of TRG and IG rearrangements* | | | | | | | | | |
| --- | --- | --- | --- | --- | --- | --- | --- | --- | --- |
| **Henrich et al. (2009)** | | | | | | | | | |
| **Primer** | **Sequence** | **Orientation** | **Labelling^1^** | | | | | | **Concentr.^2^** |
| V1FR1 | GCAAAGCATCTGGATACAGCTTC | Sense | 6-FAM | | | | | | 0.2 pmol/µl |
| V1FR3 | GAAGTTCCAGGGCAGACTCAC | Sense | 6-FAM | | | | | | 0.2 pmol/µl |
| V3FR1 | GTGGCCTCTGGATTCACCTTC | Sense | HEX | | | | | | 0.2 pmol/µl |
| V3FR3 | CCGTGAAGGGCCGATTCAC | Sense | HEX | | | | | | 0.2 pmol/µl |
| Jfam1 | CACCGTCACCAGGGCTCCTTG | Antisense |  | | | | | | 0.2 pmol/µl |
| Jfam2 | CACGGTGACCAGGGTCCCGGG | Antisense |  | | | | | | 0.2 pmol/µl |
| Jdeg | SACGGTGACYWGGGTDCCHTG | Antisense |  | | | | | | 2 pmol/µl |
| **Reaction** | **Primer combination** | **Target** |  | | | | | | |
| 1 | V1FR1 + Jfam1 + Jfam2 + Jdeg | IG |  | | | | | | |
| 2 | V1FR3 + Jfam1 + Jfam2 + Jdeg | IG |  | | | | | | |
| 3 | V3FR1 + Jfam1 + Jfam2 + Jdeg | IG |  | | | | | | |
| 4 | V3FR3 + Jfam1 + Jfam2 + Jdeg | IG |  | | | | | | |
|  | | | | | | | | | |
| **Mochizuki et al. (2011)** | | | | | | | | | |
| **Primer** | **Sequence** | **Orientation** | **Labelling^1^** | | | | | **Concentr.^2^** | |
| V1F1 | GCTGGTGCAGTCTGGGGCTG | Sense | HEX | | | | | 0.2 pmol/µl | |
| V1F2 | GCAGACACATCCACAAACACAGCCTAC | Sense | HEX | | | | | 0.2 pmol/µl | |
| V3F1 | GGTGGAGTCTGGGGGAGACCTG | Sense | 6-FAM | | | | | 0.2 pmol/µl | |
| V3F2 | GGGGGTCCCTGAGACTCACCTG | Sense | 6-FAM | | | | | 0.2 pmol/µl | |
| V3F3 | GGGTCCGCCAGGCTCCAGG | Sense | HEX | | | | | 0.2 pmol/µl | |
| V3F4 | GGCCGATTCACCATCTCCAGAGAC | Sense | 6-FAM | | | | | 0.2 pmol/µl | |
| JR1 | GCYSTCACCAGGRYTCCYBGGC | Antisense |  | | | | | 2 pmol/µl | |
| JR2 | GCTGYGACHMTDGTTCCAYGGCCC | Antisense |  | | | | | 2 pmol/µl | |
| JR3 | GCGRTGAYCWGGGTRYCYTGGC | Antisense |  | | | | | 2 pmol/µl | |
| JR4 | GCGGTGACCAGGGTCCCGGGGCCC | Antisense |  | | | | | 0.2 pmol/µl | |
| JR5 | GCCGTCACCAGGGTTCCGACGCC | Antisense |  | | | | | 0.2 pmol/µl | |
| **Reaction** | **Primer combination** | **Target** |  | | | | | | |
| 1 | V1F1 + V3F1 + JR1 + JR2 + JR3 + JR4 + JR5 | IG |  | | | | | | |
| 2 | V1F2 + V3F4 + JR1 + JR2 + JR3 + JR4 + JR5 | IG |  | | | | | | |
| 3 | V3F2 + JR1 + JR2 + JR3 + JR4 + JR5 | IG |  | | | | | | |
| 4 | V3F2 + JR1 + JR2 + JR3 + JR4 + JR5 | IG |  | | | | | | |
|  |  |  |  | | | | | | |
| **Mochizuki et al. (2012)** | | | | | | | | | |
| **Primer** | **Sequence** | **Orientation** | **Labelling^1^** | | | | **Concentr.^2^** | | |
| ftcrgv1-2 | GGSAGAAGAGCGACGAGGGCGTG | Sense |  | | | | 0.2 pmol/µl | | |
| ftcrgv3 | GGGCGAAGAGCGATGAGGGAGTG | Sense |  | | | | 0.2 pmol/µl | | |
| ftcrgv4 | GTAGTGAGGAGRATGCTGGTCTG | Sense |  | | | | 0.2 pmol/µl | | |
| ftcrgv5 | GGCAGAAGCATGACAAGGGCATG | Sense |  | | | | 0.2 pmol/µl | | |
| ftcrgj1 | CCCTGAGCAGTGTGCCAGSAC | Antisense | Atto565 | | | | 0.2 pmol/µl | | |
| ftcrgj2 | GGGGGAGTTACKATGASCTTARTTCC | Antisense | Atto550 | | | |  | | |
| **Reaction** | **Primer combination** | **Target** |  | | | | | | |
| 1 | ftcrgv1-2 + ftcrgv3 + ftcrgv4 + ftcrgv5 + ftcrgj1 | TRG |  | | | | | | |
| 2 | ftcrgv1-2 + ftcrgv3 + ftcrgv4 + ftcrgv5 + ftcrgj2 | TRG |  | | | | | | |
|  | | | | | | | | | |
| **Moore et al. (2005)** | | | | | | | | | |
| **Primer** | **Sequence** | **Orientation** | **Labelling^1^** | | | |  | | |
| TCRG V | AAGAGCGAYGAGGGMGTGT | Sense | 6-FAM | | | |  | | |
| TCRG J | CTGAGCAGTGTGCCAGSACC | Antisense |  | | | |  | | |
| **Reaction** | **Primer combination** | **Target** |  | | | | | | |
| 1 | TCRG V + TCRG J | TRG |  | | | | | | |
|  | | | | | | | | | |
| **Rout et al. (2019)** | | | | | | | | | |
| **Primer** | **Sequence** | **Orientation** | **Labelling^1^** | | | |  | | |
| TRGV-f1 | GGATGCTGGTCTGTATTACTGCG | Sense |  | | | |  | | |
| TRGV-f2 | AAGAGCGAYGAGGGMGTGT | Sense |  | | | |  | | |
| TRGJ-r1 | TGACCCTGAGCAGTGTGCCAG | Antisense | HEX | | | |  | | |
| TRGJ-r2 | GTTACGATGASCTTAGTTCCTTCTGC | Antisense | 6-FAM | | | |  | | |
| IGHV-f1 | GACACATCCACAAACACAGCC | Sense | 6-FAM | | | |  | | |
| IGHV-f2 | AAGACCGAGGACACGGCCACATAY | Sense | HEX | | | |  | | |
| IGHJ-r1 | AGGASACGGTGACCAGGG | Antisense | (6-FAM) | | | |  | | |
| IGHJ-r2 | GGGAGACGRTGACCTGGG | Antisense | (6-FAM) | | | |  | | |
| IGHJ-r3 | ACACCGTCACCAGGGCTCC | Antisense | (6-FAM) | | | |  | | |
| IGHJ-r4 | TGAGGACACTGTGACTATGGTTC | Antisense |  | | | |  | | |
| IGHJ-r5 | AGGACACTGTGACTATGGTTC | Antisense | 6-FAM | | | |  | | |
| IGHD-f1 | GGGCTTTTTGACGGGGAACTTTC | Sense |  | | | |  | | |
| IGHD-f2 | CCTGGTTATTGTCATGGGGCATC | Sense |  | | | |  | | |
| IGHD-f3 | CCCCAGCAGCCAAGAGGTTTA | Sense |  | | | |  | | |
| IGHD-f4 | TTGGTGGATTTTGTGCAGGCC | Sense |  | | | |  | | |
| IGKDE-f | GTGCGCCTAGCCACTAAAGG | Sense |  | | | |  | | |
| IGKDE-r | TTCCCAGGGGAGAATGAGTC | Antisense | HEX | | | |  | | |
| IGLV-f | CTGACTCAGCCGGCCTCAGTGTC | Sense | Atto565 | | | |  | | |
| IGLV-r1 | GACGGTCAGATGGGTMCCTC | Antisense |  | | | |  | | |
| IGLV-r2 | GACGGTCACCTKGGTCCCTC | Antisense |  | | | |  | | |
| IGLV-r3 | GATGGTCACCCGGGTCCCAT | Antisense |  | | | |  | | |
| IGLV-r4 | GACCGTCAGCCAGGTCCCTC | Antisense |  | | | |  | | |
| **Reaction** | **Primer combination** | **Target** |  | | | | | | |
| 1 | TRGV-f1 + TRGV-f2 + TRGJ-r1 + TRGJ-r2 | TRG |  | | | | | | |
| 2 | IGHV-f1 + IGHV-f2 + IGHJ-r1 +  IGHJ-r2 + IGHJ-r3 + IGHJ-r4 | IG | Note: Primer IGHJ‑r1/IGHJ‑r2/IGHJ‑r3 in this reaction unlabeled | | | | | | |
| 3 | IGHD-f1 + IGHD-f2 + IGHD-f3 + IGHD-f4 + IGHJ-r1 + IGHJ-r2 + IGHJ-r3 + IGHJ-r5  + | IG  (incompl. IGH-DJ rearrangements) | Note: Primer IGHJ‑r1/IGHJ‑r2/IGHJ‑r3 in this reaction labeled | | | | | | |
|  | IGKDE-f + IGKDE-r  + | IG  (Kde rearrangements) |  |  |  |  |  |  |  |
|  | IGLV-f + IGLV-r1 + IGLV-r2 + IGLV-r3 + IGLV-r4 | IG  (IGL rearrangements) |  |  |  |  |  |  |  |
|  | | | | | | | | | |
| **Weiss et al. (2011)** | | | | | | | | | |
| **Primer** | **Sequence** | **Orientation** | **Labelling^1^** | | |  | | | |
| FTGFR1f1 | CAGCCVGCTGTGGTGGTG | Sense |  | | |  | | | |
| FTGFR1f2 | ACAATTCGTACCATCCATTACCAAG | Sense |  | |  | | | | |
| FTGFR1f3 | TGGCGTGGCAGCGATC | Sense |  | |  | | | | |
| FTGFR2f1 | GGTCAGCTACATCCACTGGTAC | Sense |  | |  | | | | |
| FTGFR2f2 | ATCCACTGGTACCTCCACCAG | Sense |  | |  | | | | |
| FTGFR4r1 | ACCCTGAGAGTGTGCCAG | Antisense | 6-FAM | |  | | | | |
| FTGFR4r2 | GAGTTACTATGAGCTTAGTTCCTTC | Antisense | HEX | |  | | | | |
| **Reaction** | **Primer combination** | **Target** |  | | | | | | |
| 1 | FTGFR1f2 + FTGFR4r2 | TRG |  | | | | | | |
| 2a  2b | FTGFR1f3 + FTGFR4r1  FTGFR1f1 + FTGFR4r1 (seminested) | TRG |  | | | | | | |
| 3a  3b | FTGFR1f3 + FTGFR4r2  FTGFR1f1 + FTGFR4r2 (seminested) | TRG |  | | | | | | |
| 4a  4b | FTGFR2f1 + FTGFR4r1  FTGFR2f2 + FTGFR4r1 (seminested) | TRG |  | | | | | | |
| 5a  5b | FTGFR2f1 + FTGFR4r2  FTGFR2f2 + FTGFR4r2 (seminested) | TRG |  | | | | | | |
|  |  |  |  | | | | | | |
| **Werner et al. (2005)** | | | | | | | | | |
| **Primer** | **Sequence** | **Orientation** | **Labelling^1^** |  | | | | | |
| FR2 | CCAGGCTCCAGGGAAGGG | Sense |  |  | | | | | |
| FR3 | TCCAGAGACAACGCCAAGAAC | Sense |  |  | | | | | |
| J2 | TGAGGACACTGTGACTATGGTTCC | Antisense | 6-FAM |  | | | | | |
| JD | GGACACCGTCACYAKGVYTCC | Antisense | Atto532 |  | | | | | |
| **Reaction** | **Primer combination** | **Target** |  | | | | | | |
| 1 | FR2 + J2 | IG |  | | | | | | |
| 2 | FR2 + JD | IG |  | | | | | | |
| 3 | FR3 + J2 | IG |  | | | | | | |
| 4 | FR3 + JD | IG |  | | | | | | |
| *Primers considered by the authors as unnecessary for their diagnostic system are omitted.  ^1^Labelling of primers may differ from the reported labelling.  ^2^Final concentration in 25 μl PCR reaction | | | | | | | | | |

| **Supplementary table 2:** Cycling conditions for the analysis of IG and TRG rearrangements |
| --- |

| Step | **Henrich et al. (2009)** | | | | **Mochizuki et al. (2011/12)** | | | | | | | **Moore et al. (2005)** | | | | | | **Rout et al. (2019)** | | | | | | | **Werner et al. (2009)** | | | | |
| --- | --- | --- | --- | --- | --- | --- | --- | --- | --- | --- | --- | --- | --- | --- | --- | --- | --- | --- | --- | --- | --- | --- | --- | --- | --- | --- | --- | --- | --- |
|  | T* | | t^#^ | | T | | | | t | | | T | | | t | | | T | | | t | | | | T | | | t | |
|  |  | |  | |  | | | |  | | |  | | |  | | |  | | |  | | | |  | | |  | |
| 1. Initial activation | 95 | | 300 | | 95 | | | | 300 | | | 95 | | | 300 | | | 95 | | | 300 | | | | 95 | | | 300 | |
| 2. Melting | 92 | | 15 | | 95 | | | | 30 | | | 94 | | | 30^1,2^/20^3^ | | | 94 | | | 30 | | | | 94 | | | 30 | |
| 3. Annealing | 68.5 | | 90 | | 68 | | | | 90 | | | 70^1^/68^2^/65^3^ | | | 60 | | | 64-59^4^/59^5^ | | | 30 | | | | 70^6^/68^7^/65^8^ | | | 120 | |
| 4. Elongation | 72 | | 10 | | 72 | | | | 30 | | | - | | | - | | | 72 | | | 90 | | | | - | | | - | |
| 5. Final extension | 72 | | 600 | | 72 | | | | 1800 | | | 72 | | | 600 | | | 72 | | | 600 | | | | 72 | | | 600 | |
| Numbers of cycles | 2.‑4.: 35 | | | | 2.‑4.: 40 | | | | | | | 2.-3.: ^1^5 + ^2^5 + ^3^35 | | | | | | 2.‑4.: ^4^10 (-0.5 °C/cycle) + ^5^30 | | | | | | | 2.-3.: ^6^5 + ^7^5 + ^8^35 | | | | |
|  |  | | | | | |  | | | | | | | | |  | | | | | | |  | | | | | | |
| Step | **Weiss et al. (2009)** | | | | | | | | | | | | | | | | | | | | | | | | | | | | |
|  |  | R1^10^ | | | | R2a | | R2b | | | R3a | | | R3b | | | | R4a | | R4b | | | | R5a | | | R5b | | |
|  | t | T | | n^9^ | | T | n | T | | n | T | | n | T | | | n | T | n | T | | n | | T | | n | T | | n |
|  |  |  | |  | |  |  |  | |  |  | |  |  | | |  |  |  |  | |  | |  | |  |  | |  |
| 1. Initial activation | 300 | 95 | |  | | 95 |  | 95 | |  | 95 | |  | 95 | | |  | 95 |  | 95 | |  | | 95 | |  | 95 | |  |
| 2. Melting | 30 | 92 | | 10 | | 92 | 10 | 92 | | 12 | 92 | | 12 | 92 | | | 10 | 92 | 10 | 92 | | 10 | | 92 | | 12 | 92 | | 10 |
| 3. Annealing | 15 | 62^11^ | |  | | 72^12^ |  | 72^11^ | |  | 72^11^ | |  | 65^11^ | | |  | 72^13^ |  | 72^11^ | |  | | 68^11^ | |  | 65^11^ | |  |
| 4. Elongation | 45/30^14^ | 72 | |  | | 72 |  | 72 | |  | 72 | |  | 72 | | |  | 72 |  | 72 | |  | | 72 | |  | 72 | |  |
| 5. Melting | 30 | 92 | | 30 | | 92 | 10 | 92 | | 22 | 92 | | 10 | 92 | | | 24 | 92 | 10 | 92 | | 24 | | 92 | | 10 | 92 | | 24 |
| 6. Annealing | 15 | 57 | |  | | 68 |  | 66 | |  | 66 | |  | 60 | | |  | 69 |  | 66 | |  | | 62 | |  | 60 | |  |
| 7. Elongation | 45/30^14^ | 72 | |  | | 72 |  | 72 | |  | 72 | |  | 72 | | |  | 72 |  | 72 | |  | | 72 | |  | 72 | |  |
| 8. Melting | 30 | - | | - | | 92 | 20 | - | | - | 92 | | 20 | - | | | - | 92 | 20 | - | | - | | 92 | | 20 | - | | - |
| 9. Annealing | 15 | - | |  | | 66 |  | - | |  | 74 | |  | - | | |  | 67 |  | - | |  | | 60 | |  | - | |  |
| 10. Elongation | 30 | - | |  | | 72 |  | - | |  | 72 | |  | - | | |  | 72 |  | - | |  | | 72 | |  | - | |  |
| 11. Final extension | 600 | 72 | |  | | 72 |  | 72 | |  | 72 | |  | 72 | | |  | 72 |  | 72 | |  | | 72 | |  | 72 | |  |
| ^9^n = number of cycles (repeated steps between dashed lines); ^10^R1 = reaction 1, 2, etc. (see suppl. table 1)  ^11^-0.5 °C per cycle; ^12^-0.4 °C per cycle; ^13^ - 0.3 °C per cycle  ^14^30 °C in R2b, R3b, R4b, R5b | | | | | | | | | | | | | | | | | | | | | | | | | | | | | |
| ^*^T = temperature in °C; ^#^t = time in seconds | | | | | | | | | | | | | | | | | | | | | | | | | | | | | |

| **Supplementary table 3:** Results of TRG clonality analysis | | | | |
| --- | --- | --- | --- | --- |
| Sample | Moore et al. | Mochizuki et al. | Weiss et al. | Rout et al. |
| T-cell lymphomas | | | | |
| 1 | ✓ | ✓ | ✓ | ✓ |
| 2 | ✓ | ✓ | -- | ✓ |
| 3 | 🗶 | 🗶 | -- | 🗶 |
| 4 | ✓ | ✓ | -- | ✓ |
| 5 | ✓ | ✓ | ✓ | ✓ |
| 6 | 🗶 | 🗶 | ✓ | ✓ |
| 7 | 🗶 | ✓ | -- | ✓ |
| 8 | ✓ | ✓ | ✓ | ✓ |
| 9 | ✓ | ✓ | -- | ✓ |
| 10 | ✓ | ✓ | ✓ | ✓ |
| 11 | ✓ | ✓ | ✓ | ✓ |
| 12 | 🗶 | ✓ | ✓ | ✓ |
| 13 | ✓ | ✓ | ✓ | ✓ |
| 14 | 🗶 | ✓ | ✓ | 🗶 |
| 15 | ✓ | ✓ | ✓ | ✓ |
| 16 | 🗶 | ✓ | 🗶 | 🗶 |
| 17 | ✓ | ✓ | ✓ | ✓ |
| 18 | ✓ | ✓ | 🗶 | ✓ |
| 19 | 🗶 | 🗶 | 🗶 | 🗶 |
| 20 | 🗶 | 🗶 | ✓ | 🗶 |
| 21 | ✓ | ✓ | ✓ | ✓ |
| 22 | ✓ | ✓ | ✓ | ✓ |
| 23 | 🗶 | ✓ | ✓ | ✓ |
| 24 | 🗶 | 🗶 | 🗶 | 🗶 |
| 25 | 🗶 | 🗶 | 🗶 | 🗶 |
| 26 | 🗶 | ✓ | 🗶 | 🗶 |
| 27 | 🗶 | ✓ | ✓ | ✓ |
| 28 | ✓ | ✓ | ✓ | ✓ |
| 29 | 🗶 | 🗶 | 🗶 | 🗶 |
| 30 | 🗶 | ✓ | ✓ | ✓ |
| 31 | 🗶 | 🗶 | 🗶 | 🗶 |
| Polyclonal control group | | | | |
| 61 | 🗶 | 🗶 | 🗶 | 🗶 |
| 62 | 🗶 | 🗶 | 🗶 | 🗶 |
| 63 | 🗶 | 🗶 | 🗶 | 🗶 |
| 64 | 🗶 | 🗶 | 🗶 | 🗶 |
| 65 | 🗶 | 🗶 | 🗶 | 🗶 |
| 66 | 🗶 | 🗶 | 🗶 | 🗶 |
| 67 | 🗶 | 🗶 | 🗶 | 🗶 |
| 68 | 🗶 | 🗶 | 🗶 | 🗶 |
| 69 | ✓ | ✓ | ✓ | 🗶 |
| 70 | 🗶 | 🗶 | 🗶 | 🗶 |
| 71 | 🗶 | 🗶 | 🗶 | 🗶 |

| **Supplementary table 4:** Results of IG clonality analysis | | | | |
| --- | --- | --- | --- | --- |
| Sample | Werner et al. | Henrich et al. | Mochizuki et al. | Rout et al. |
| B-cell lymphomas | | | | |
| 32 | 🗶 | ✓ | ✓ | ✓ |
| 33 | 🗶 | 🗶 | 🗶 | 🗶 |
| 34 | ✓ | ✓ | ✓ | ✓ |
| 35 | ✓ | ✓ | ✓ | (✓) |
| 36 | ✓ | 🗶 | ✓ | ✓ |
| 37 | 🗶 | 🗶 | 🗶 | ✓ |
| 38 | ✓ | ✓ | ✓ | ✓ |
| 39 | 🗶 | 🗶 | 🗶 | 🗶 |
| 40 | 🗶 | 🗶 | 🗶 | ✓ |
| 41 | ✓ | ✓ | ✓ | ✓ |
| 42 | 🗶 | 🗶 | ✓ | (✓) |
| 43 | 🗶 | ✓ | ✓ | ✓ |
| 44 | 🗶 | 🗶 | 🗶 | 🗶 |
| 45 | 🗶 | 🗶 | ✓ | 🗶 |
| 46 | 🗶 | 🗶 | 🗶 | 🗶 |
| 47 | ✓ | ✓ | ✓ | ✓ |
| 48 | 🗶 | 🗶 | ✓ | ✓ |
| 49 | ✓ | ✓ | ✓ | ✓ |
| 50 | 🗶 | 🗶 | 🗶 | 🗶 |
| 51 | 🗶 | 🗶 | 🗶 | (✓) |
| 52 | 🗶 | 🗶 | 🗶 | 🗶 |
| 53 | 🗶 | 🗶 | 🗶 | (✓) |
| 54 | 🗶 | 🗶 | ✓ | 🗶 |
| 55 | 🗶 | ✓ | ✓ | ✓ |
| 56 | 🗶 | 🗶 | ✓ | ✓ |
| 57 | 🗶 | 🗶 | 🗶 | ✓ |
| 58 | ✓ | ✓ | ✓ | ✓ |
| 59 |  | ✓ | ✓ | ✓ |
| 60 | ✓ | ✓ | ✓ | ✓ |
| Polyclonal control group | | | | |
| 61 | 🗶 | 🗶 | 🗶 | 🗶 |
| 62 | 🗶 | 🗶 | 🗶 | 🗶 |
| 63 | 🗶 | 🗶 | 🗶 | 🗶 |
| 64 | 🗶 | 🗶 | 🗶 | (✓) |
| 65 | 🗶 | 🗶 | 🗶 | ✓ |
| 66 | 🗶 | 🗶 | 🗶 | (✓) |
| 67 | 🗶 | 🗶 | 🗶 | ✓ |
| 68 | 🗶 | 🗶 | 🗶 | (✓) |
| 69 | 🗶 | 🗶 | ✓ | 🗶 |
| 70 | 🗶 | 🗶 | 🗶 | (✓) |
| 71 | 🗶 | 🗶 | ✓ | 🗶 |

| ✓: | clonality detected | 🗶: | no clonality detected | (✓): | clonality only with IGKDE-PCR |
| --- | --- | --- | --- | --- | --- |

| **Supplementary table 5:** Results of DNA quality analysis | | | | | |
| --- | --- | --- | --- | --- | --- |
| Sample | A260/A280 | A260/A230 | 150 bp | 188 bp | 300 bp |
| T cell lymphoma | | | | | |
| 1 | 1,85 | 2,22 | **+** | **/** | **+** |
| 2 | 1,72 | 2,17 | **+** | **+** | **-** |
| 3 | 1,75 | 2,02 | **+** | **+** | **-** |
| 4 | 1,73 | 1,91 | **+** | **+** | **-** |
| 5 | 1,8 | 2,07 | **+** | **/** | **+** |
| 6 | 1,8 | 2,13 | **+** | **/** | **+** |
| 7 | 1,69 | 1,5 | **+** | **+** | **-** |
| 8 | 1,82 | 2,11 | **+** | **/** | **+** |
| 9 | 1,75 | 1,9 | **+** | **+** | **+** |
| 10 | 1,78 | 2,11 | **+** | **/** | **+** |
| 11 | 1,88 | 2,25 | **+** | **/** | **+** |
| 12 | 1,91 | 2,28 | **+** | **/** | **+** |
| 13 | 1,77 | 1,83 | **+** | **+** | **-** |
| 14 | 1,82 | 1,98 | **+** | **+** | **-** |
| 15 | 1,85 | 2,08 | **+** | **/** | **+** |
| 16 | 1,87 | 2,14 | **+** | **/** | **+** |
| 17 | 1,84 | 2,19 | **+** | **/** | **+** |
| 18 | 1,82 | 1,89 | **+** | **/** | **+** |
| 19 | 1,81 | 2,17 | **+** | **/** | **+** |
| 20 | 1,85 | 2,06 | **+** | **/** | **+** |
| 21 | 1,77 | 1,9 | **+** | **/** | **+** |
| 22 | 1,85 | 1,66 | **+** | **/** | **+** |
| 23 | 1,78 | 2,09 | **+** | **/** | **+** |
| 24 | 1,82 | 2,07 | **+** | **/** | **+** |
| 25 | 1,84 | 1,9 | **+** | **/** | **+** |
| 26 | 1,84 | 2,17 | **+** | **/** | **+** |
| 27 | 1,85 | 1,84 | **+** | **/** | **+** |
| 28 | 1,82 | 2,08 | **+** | **/** | **+** |
| 29 | 1,82 | 2,01 | **+** | **/** | **+** |
| 30 | 1,77 | 2,1 | **+** | **/** | **+** |
| 31 | 1,79 | 1,61 | **+** | **/** | **+** |
| B cell lymphoma | | | | | |
| 32 | 1,79 | 2,14 | **+** | **/** | **+** |
| 33 | 1,85 | 2,19 | **+** | **/** | **+** |
| 34 | 1,86 | 2,13 | **+** | **/** | **+** |
| 35 | 1,87 | 2,13 | **+** | **/** | **+** |
| 36 | 1,86 | 2,16 | **+** | **/** | **+** |
| 37 | 1,86 | 2,23 | **+** | **/** | **+** |
| 38 | 1,8 | 1,91 | **+** | **/** | **+** |
| 39 | 1,83 | 2,18 | **+** | **/** | **+** |
| 40 | 1,76 | 1,93 | **+** | **+** | **-** |
| 41 | 1,81 | 2,19 | **+** | **/** | **+** |
| 42 | 1,84 | 2,19 | **+** | **/** | **+** |
| 43 | 1,85 | 2,15 | **+** | **/** | **+** |
| 44 | 1,79 | 2,02 | **+** | **+** | **-** |
| 45 | 1,78 | 2,12 | **+** | **/** | **+** |
| 46 | 1,81 | 2,19 | **+** | **/** | **+** |
| 47 | 1,75 | 2,09 | **+** | **+** | **-** |
| 48 | 1,81 | 2,08 | **+** | **/** | **+** |
| 49 | 1,83 | 1,73 | **+** | **/** | **+** |
| 50 | 1,79 | 1,88 | **+** | **+** | **-** |
| 51 | 1,82 | 2,0 | **+** | **/** | **+** |
| 52 | 1,8 | 1,75 | **+** | **+** | **+** |
| 53 | 1,83 | 2,06 | **+** | **/** | **+** |
| 54 | 1,79 | 2,1 | **+** | **+** | **-** |
| 55 | 1,8 | 2,06 | **+** | **/** | **+** |
| 56 | 1,81 | 1,92 | **+** | **/** | **+** |
| 57 | 1,8 | 1,98 | **+** | **/** | **+** |
| 58 | 1,83 | 1,97 | **+** | **/** | **+** |
| 59 | 1,72 | 2,04 | **+** | **-** | **-** |
| 60 | 1,82 | 2 | **+** | **/** | **+** |
| Polyclonal control samples | | | | | |
| 61 | 1,77 | 2,17 | **+** | **/** | **+** |
| 62 | 1,8 | 2,2 | **+** | **+** | **-** |
| 63 | 1,81 | 1,95 | **+** | **/** | **+** |
| 64 | 1,85 | 2,19 | **+** | **/** | **+** |
| 65 | 1,85 | 2,28 | **+** | **/** | **+** |
| 66 | 1,83 | 2,15 | **+** | **/** | **+** |
| 67 | 1,81 | 1,84 | **+** | **/** | **+** |
| 68 | 1,84 | 2,09 | **+** | **/** | **+** |
| 69 | 1,83 | 2,17 | **+** | **/** | **+** |
| 70 | 1,84 | 2,23 | **+** | **/** | **+** |
| 71 | 1,8 | 2,18 | **+** | **/** | **+** |

| **Supplementary table 6:** Expected size ranges | | |
| --- | --- | --- |
| **Primer combination** | **Original publication** | **Calculated in this study** |
| **T cell lymphoma** | | |
| **Moore et al., 2005** |  |  |
|  | 80 to 120 bp | 61 to 136 bp |
| **Weiss et al., 2011** |  |  |
| FR1f2-PCR | 325 to 350 bp | 305 to 385 bp |
| FR1f3/FR4r1 semi-nested PCR | 325 to 350 bp | 320 to 372 bp |
| FR1f3/FR4r2 semi-nested PCR | 325 to 350 bp | 310 to 360 bp |
| FR2f1/FR4r1 semi-nested PCR | 250 to 280 bp | 241 to 299 bp |
| FR2f1/FR4r2 semi-nested PCR | 250 to 280 bp | 228 to 289 bp |
| **Mochizuki et al., 2012** |  |  |
| J1 PCR | 80 to 120 bp | 85 to 136 bp |
| J2 PCR | 80 to 120 bp | 79 to 132 bp |
| **Rout et al., 2019** |  |  |
| TRGJ-r1-Reaktion | 80 to 125 bp | 74 to 138 bp |
| TRGJ-r2-Reaktion | 70 to 105 bp | 55 to 111 bp |
| **B cell lymphoma** | | |
| **Werner et al., 2005** |  |  |
| FR2/J2 PCR | 250 to 300 bp | 225 to 289 bp |
| FR2/JD PCR | 250 to 300 bp | 217 to 285 bp |
| FR3/J2 PCR | 130 to 180 bp | 126 to 194 bp |
| FR3/JD PCR | 130 to 180 bp | 119 to 195 bp |
| **Henrich et al., 2009** |  |  |
| V1FR3 PCR | 150 to 200 bp | 142 to 198 bp |
| V3FR3 PCR | 150 to 200 bp | 129 to 231 bp |
| V1FR1 PCR | 300 to 350 bp | not calculated |
| V3FR1 PCR | 300 to 350 bp | 254 to 330 bp |
| **Mochizuki et al., 2011** |  |  |
| V1F1 PCR | 310 to 380 bp | 318 to 378 bp |
| V3F1 PCR | 310 to 380 bp | 309 to 385 bp |
| V3F4 PCR | 120 to 190 bp | 120 to 200 bp |
| V1F2 PCR | 100 to 170 bp | 111 to 179 bp |
| V3F2 PCR | 270 to 340 bp | 274 to 362 bp |
| V3F3 PCR | 210 to 280 bp | 217 to 297 bp |
| **Rout et al., 2019** |  |  |
| IGHV-f1 PCR | 103 to 175 bp | 110 to 170 bp |
| IGHV-f2 PCR | 57 to 153 bp | 70 to 130 bp |
| IGHV incomplete | not calculated | 65 to 110 bp |
| IGKDE PCR | 177 to 201 bp | 180 to 200 bp |
| IGL PCR | 283 to 347 bp | 300 to 340 bp |
